# Five Days Granulocyte Colony-Stimulating Factor Treatment Increases Bone Formation and Reduces Gap Size of a Rat Segmental Bone Defect: A Pilot Study

**DOI:** 10.3389/fbioe.2018.00005

**Published:** 2018-02-12

**Authors:** Marietta Herrmann, Stephan Zeiter, Ursula Eberli, Maria Hildebrand, Karin Camenisch, Ursula Menzel, Mauro Alini, Sophie Verrier, Vincent A. Stadelmann

**Affiliations:** ^1^AO Research Institute Davos, Davos, Switzerland

**Keywords:** critical size bone defect, fracture, bone regeneration, endothelial progenitor cells, vascularization, granulocyte colony-stimulating factor, cell mobilization

## Abstract

Bone is an organ with high natural regenerative capacity and most fractures heal spontaneously when appropriate fracture fixation is provided. However, additional treatment is required for patients with large segmental defects exceeding the endogenous healing potential and for patients suffering from fracture non-unions. These cases are often associated with insufficient vascularization. Transplantation of CD34+ endothelial progenitor cells (EPCs) has been successfully applied to promote neovascularization of bone defects, however including extensive *ex vivo* manipulation of cells. Here, we hypothesized, that treatment with granulocyte colony-stimulating factor (G-CSF) may improve bone healing by mobilization of CD34+ progenitor cells into the circulation, which in turn may facilitate vascularization at the defect site. In this pilot study, we aimed to characterize the different cell populations mobilized by G-CSF and investigate the influence of cell mobilization on the healing of a critical size femoral defect in rats. Cell mobilization was investigated by flow cytometry at different time points after five consecutive daily G-CSF injections. In a pilot study, bone healing of a 4.5-mm critical femoral defect in F344 rats was compared between a saline-treated control group and a G-CSF treatment group. *In vivo* microcomputed tomography and histology were applied to compare bone formation in both treatment groups. Our data revealed that leukocyte counts show a peak increase at the first day after the last G-CSF injection. In addition, we found that CD34+ progenitor cells, including EPCs, were significantly enriched at day 1, and further increased at day 5 and day 11. Upregulation of monocytes, granulocytes and macrophages peaked at day 1. G-CSF treatment significantly increased bone volume and bone density in the defect, which was confirmed by histology. Our data show that different cell populations are mobilized by G-CSF treatment in cell specific patterns. Although in this pilot study no bridging of the critical defect was observed, significantly improved bone formation by G-CSF treatment was clearly shown.

## Introduction

While the majority of bone fractures and bone defects heal, nonunion is a common complication of a fracture; it indicates that fracture healing is not happening in a timely fashion. For example, nonunion occurs in about 10% of all tibia fractures (Antonova et al., 2013) requiring additional interventions. A recent study analyzing about 5,000 fracture cases in Scotland, revealed a general non-union rate of 1.9% which is much lower than previously believed. On the other hand, it was also reported that for certain fracture types the non-union rate rose up to 9% (Mills et al., 2017). Similarly, there exist many strategies with varying degrees of success for the posttraumatic management of large segmental defects. In clinical practice, the gold standard is still autogenous or allogenic bone grafting, but these come with obvious drawbacks such as comorbidities and limited material availability (Giannoudis et al., 2011). Bone transport methods such as Ilizarov have proven popular for some time but they require extensive care and are not exempt from complications (Catagni et al., 2011). Pharmacologic options such as BMP2 and PTH can improve bone healing to a certain degree, but it has been shown that bone regenerates better in presence of a mechanical support, such as a scaffold, for the cells to migrate on (Lichte et al., 2011). The research community is thus massively investigating into tissue engineered constructs with successes and failures (Keating et al., 2005; Hulsart-Billstrom et al., 2016).

Independently of the treatment strategy, a hallmark of failure to heal is an insufficient vascular supply which leads to hypoxia and reduced nutrient availability at the site of injury. Various treatment strategies have focused on supporting neovascularization within the fracture gap, for example, by local administration of proangiogenic growth factors such as HIF-1α (Stewart et al., 2011) or vascular endothelial growth factor (Herrmann et al., 2015). Alternatively, transplantation of endothelial cells or endothelial progenitor cells (EPCs) are under investigation and have been extensively reviewed elsewhere (Rao and Stegemann, 2013; Sun et al., 2016; Kawakami et al., 2017).

Endothelial progenitor cells have first been discovered in the CD34+ hematopoietic progenitor cell (HPC) population in peripheral blood (Asahara et al., 1997). CD34+ cells, enriched from blood or bone marrow, were successfully transplanted to treat ischemic diseases (Kalka et al., 2000; Kawamoto et al., 2003; Iwasaki et al., 2006) as well as to support vascularization in tissue engineered constructs (Rozen et al., 2009; Atesok et al., 2010; Seebach et al., 2012; Herrmann et al., 2014; Bates et al., 2017; Giles et al., 2017; Liu et al., 2017a; Nau et al., 2017). Aforementioned studies have focused on local cell transplantation. Only few studies have investigated a systemic transplantation of CD34+ cells addressing the natural homing capacity of these cells (Matsumoto et al., 2006; Terayama et al., 2011). Due to the limited availability of cells, most approaches have used *ex vivo* expanded cells. This is associated with several drawbacks including long expansion times, costs and safety issues which arise upon manipulation of cells.

Granulocyte colony-stimulating factor (G-CSF) is an important mediator of granulopoiesis. G-CSF-deficient mice suffer from neutropenia and impaired mobilization of neutrophils in the blood (Lieschke et al., 1994). In clinics, G-CSF and biosimilars are used to treat patients with neutropenia during intensive chemotherapy and for mobilization of hematopoietic stem cells in the circulation (Gazitt, 2002; Mehta et al., 2015; Hsu and Cushing, 2016).

With the discovery of EPCs in the CD34+ HPC fraction (see above), G-CSF became of interest for the treatment of diseases involving impaired vascularization. G-CSF treatment can be applied to increase the frequency of EPCs in the circulation and by this to improve the yield of donor cells for transplantation approaches. For the treatment of critical limb ischemia, a phase I/IIa clinical trials has been performed to assess transplantation of G-CSF mobilized cells and suggested safety and feasibility of this approach (Kawamoto et al., 2009). In the field of bone regeneration, Kuroda et al. (2011, 2014b) reported on beneficial effects of transplantation of G-CSF mobilized CD34+ cells in nonunion patients. The application of G-CSF mobilized EPCs in orthopedics has also been addressed in a recent review (Kawakami et al., 2017).

Beside for cell therapies, G-CSF has also been utilized to booster the mobilization of endogenous cells. This was first demonstrated in the context of cardiovascular and ischemic diseases and was recently reviewed (D’Amario et al., 2017). It was shown that systemic G-CSF administration promoted reendothelialization in a mouse model of vascular injury (Yoshioka et al., 2006) as well as vascularization in hindlimb ischemia (Capoccia et al., 2006; Jeon et al., 2006). In addition, drug-delivery and tissue engineering approaches have focused on the local delivery of G-CSF to the respective defect site in the context of wound healing (Tanha et al., 2017), hindlimb ischemia (Layman et al., 2009) and chronic myocardial infarction (Spadaccio et al., 2017). In the context of bone regeneration, Ishida et al. (2010) demonstrated that treatment of a segmental bone defect in the rabbit ulna with a G-CSF loaded gelatin hydrogel resulted in accelerated bone formation. In line with this, it was shown that local delivery of G-CSF to osteoporotic bone fractures (Liu et al., 2017b) and a rat calvarial defect (Minagawa et al., 2014) promoted new bone formation in both models. Assuming that an enhanced accumulation of stem cells in the circulation would facilitate their homing capacity, Marmotti et al. (2013) investigated the effect of preoperative administration of G-CSF in patients undergoing high tibial valgus osteotomy with bone graft substitution. This preliminary clinical study suggested that G-CSF pretreatment might accelerate the integration of graft material (Marmotti et al., 2013).

While most of the aforementioned studies have addressed either transplantation of G-CSF-mobilized cells or local delivery of G-CSF, we were here interested to investigate systemically administered G-CSF and hypothesized that stimulation of CD34+ progenitor cells by G-CSF might promote the healing of large bone defects. First, we aimed to characterize the time course and composition of the G-CSF mobilized cell population. Finally, we performed a pilot study to test the influence of G-CSF administration on the healing of a critical size femoral defect in rats.

## Materials and Methods

### Animal Experiments

All procedures were performed in accordance with the Swiss Animal Protection Law (2014_30F; 2012_36) in an Association for Assessment and Accreditation of Laboratory Animal Care Internal (AAALAC International) approved facility. Animals were group-housed and received standard diet (Extrudat 3436, Provimi-Kliba) and water ad libidum. Adult specific pathogen free female F344 Fisher rats were obtained from Charles River (Germany). Animals were healthy based on health certificates provided by the breeder and clinical examination upon study entry.

### G-CSF Mobilized Cell Population

**Figure 1:**
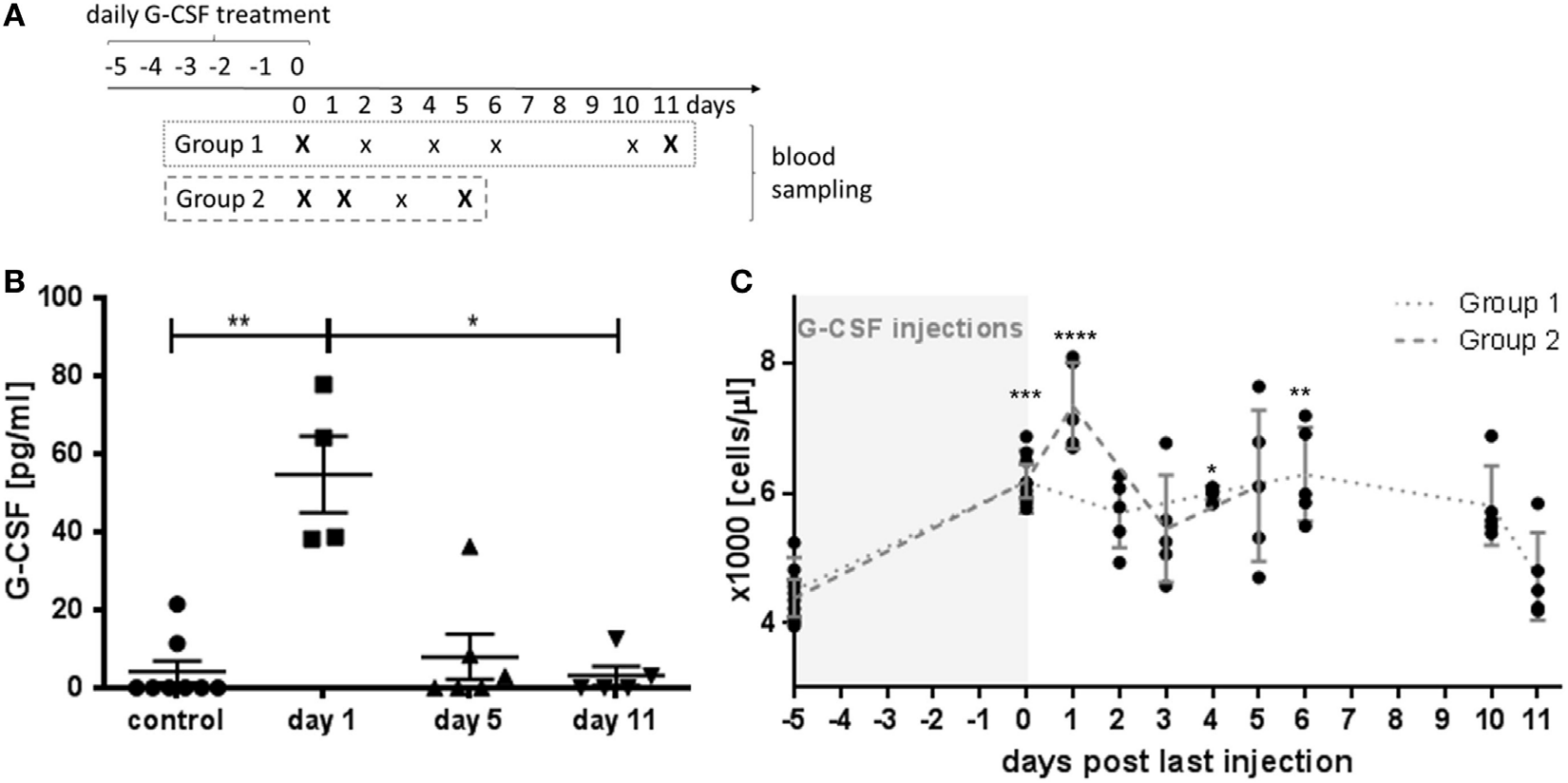
Granulocyte colony-stimulating factor (G-CSF) pharmacokinetics and time course of leukocyte stimulation. **(A)** Schematic presentation of the study design. For blood analysis, animals were randomly assigned to two different experimental groups with blood sampling at different time points after 5 days of G-CSF injections as indicate. Capital letters indicate time points of serum sampling. **(B)** Serum levels of G-CSF were determined by ELISA in untreated control animals and at indicated time points after the last G-CSF injection. **(C)** Leukocyte counts after G-CSF treatment. Note that highest leukocyte counts were observed at the first day after the last G-CSF injection. Values are given as mean ± SEM. *p < 0.05; **p < 0.01; ***p < 0.001; ****p < 0.0001 calculated using Kruskal–Wallis test with Dunn’s multiple comparison.

Twenty-nine female rats (age: 19 ± 1 weeks, 156–187 g) were used to assess the time course and composition of the G-CSF mobilized cell population and randomly assigned to five experimental groups (Figure 1A). Animals received daily subcutaneous (sc) injections of 50 µg/kg bodyweight G-CSF [Filgrastim, Teva Pharma; 50 µg/ml in 5% glucose solution (Braun)] for 5 consecutive days, which refers to a previously described treatment protocol (Kong et al., 2004; Shyu et al., 2004). Prior to injection, animals were lightly sedated (<5 min) with isofluran (1–2 vol%, Baxter AG). 30–100 µl of blood was collected from the tail vein to measure leukocyte counts using VetABC hematology analyzer (Medical Solution GmbH). At study end points, animals were anesthetized with isoflurane, the whole blood collected by heart puncture and animals euthanatized by an overdose of pentobarbital (Esconarkon^®^, Streuli Pharma AG).

### Pilot Study on the Effect of G-CSF Treatment on Bone Formation in a Critical Size Segmental Femoral Defect

In a pilot study investigating the effect of G-CSF treatment on bone formation, 10 skeletally mature female rats (age: 23.1 ± 0.2 weeks, 186–226 g) were randomly divided into two groups. Animals were injected subcutaneously once daily for 5 consecutive days after surgery with G-CSF (50 µg/kg) or saline, respectively (Figure 3A). At the day of surgery, 30–100 µl blood was collected from the tail vein to determine leukocyte counts. Rats were premedicated with Carprofen (5 mg/kg, sc, Rimadyl, Pfizer) and Buprenorphine (0.1 mg/kg, sc, Temgesic^®^, Rechitt Benckiser). Under isoflurane anesthesia, the left femur was aseptically prepared and a skin incision was made. The sc fascia lata was incised and the M. quadriceps and M. biceps femoris were separated bluntly to expose the femur. The PEEK fixation plate (RIS.602.100) was fixed on the femur, with the center of the plate at the distal level of the lateral femoral crest using six self-cutting locking screws (RIS.402.120) after predrilling the screw holes (RIS.592.202). Facilitated by a jig (RIS.302.104), a 4.5 mm defect was created by two osteotomies using a Gigly saw (RIS.590.110, all RISystem AG). Finally, fascia and skin were closed in a continuous pattern using absorbable suture material (Vicryl rapide, 5-0, Ethicon Inc., Johnson & Johnson Medical). To prevent hypothermia, a heated mat during surgery and an infrared lamp during the recovery period (approx. 30 min) were used. All animals received 3 ml Ringer’s solution (Braun) intraperitoneally. Postoperative analgesia consisted of Buprenorphine (0.1 mg/kg s.c.; 12/24 h post-OP) and Paracetamol (Dafalgan Sirup, Bristol-Myers Squibb SA; 7 ml/100 ml in drinking water; for 3 days post-OP). Animals recovered rapidly after surgery and started normal load-bearing within 30 min. All animals lost weight postoperatively (<5%) but recovered within 10 days. The animals were kept in the study for as long as bone was forming in the defect [according to microcomputed tomography (microCT)]. Once plateauing, the study was stopped (230 days).

### *In vivo* microCT

New bone volume within the defect and gap size was monitored using *in vivo* microCT (VivaCT40, Scanco Medical). The animals were scanned under isoflurane anesthesia at the indicated time points. They were placed in a modified holder in lateral recumbency. The operated leg was positioned to align the femur with the scanner axis, then fixed with masking tape to minimize metal and motion artifacts. A 8 mm region, centered on the defect, was scanned with 200 ms integration time and 1,500 projections per rotation. The X-ray tube was operated at 70 kV tension and 114 µA current. The projections were then reconstructed across an image matrix of 2,048 × 2,048 pixels with an isotropic voxel size of 19 µm. Scans showing evidence of motion artifacts were repeated. The postoperative scans were rotated to align the femur longitudinally and the screws sagittally. The subsequent scans were registered to their respective postoperative scan using rigid registration (Boyd et al., 2006).

The region of interest (ROI) for analysis was defined as the defect volume on the postoperative scan. The ROI was propagated to the registered scans. Scans were Gaussian-filtered (sigma = 0.8 support = 1) to reduce noise, bone was segmented (threshold = 515 mgHA/ccm); bone volume and density were computed within the ROI using direct voxel counting methods. The gap size was then defined as the smallest distance found in the distance map (Hildebrand and Rüegsegger, 1997) of the empty space. All image processing and analysis were performed with Image Processing Language (Scanco Medical). The intervals between scans were adapted (in round number of weeks) during the study in function of the bone formation rate (keeping ~ 0.1 mm^3^ difference between time points).

The contralateral femoral midshafts were also microCT scanned immediately postmortem using the same scan settings as described above. Cortical bone parameters such as bone density, moment of inertia, and cortical thickness were computed using the standard methods.

### Tissue Harvesting, Fixation and Histology

After 230 days, animals were euthanized with an overdose of CO_2_. Both femora were fixed for two months in 70% ethanol. After fixation, the operated femora were embedded in methyl methacyrlate (Sigma) and stained with Giemsa/eosin as described before (Rochford et al., 2016).

### ELISA Quantification of G-CSF in Serum

An ELISA (Quantikine, R&D) was used according to the manufacturer’s protocol to determine the concentration of human G-CSF in rat serum collected upon euthanasia. Absorbance was measured using Viktor3 Plate Reader (Perkin Elmer).

### Flow Cytometry Analysis of Blood Samples

Anticoagulated blood was diluted in phosphate-buffered saline (PBS) and overlaid on Ficoll density medium (1.083 g/l, Sigma-Aldrich). After centrifugation at 800 *g*, mononuclear cells (MNCs) were collected from the interphase and washed twice [PBS/5% fetal bovine serum (FBS, Pan)]. Cell counts were determined using an automated cell counter (Scepter, Millipore). For cell surface marker staining, 1 × 10^5^ MNCs were washed with buffer (PBS/1% FBS) and incubated with CD32 antibody (BD) for 5 min on ice to reduce unspecific antibody binding. Afterward cells were stained for 30 min on ice with the following antibodies: CD45-PeCy5 (ThermoFisher Scientific), CD34-APC (Antibodies Online), CD11b-FITC (BD). Unstained and isotype controls (mouse IgG1-PeCy5 (ThermoFisher), rabbit IgG-Alexa647 (Bioconcept), mouse IgG2a, k-FITC (BD)) were performed. Antibodies were used according to the manufacturer’s recommendations. To investigate LDL uptake, 1 × 10^5^ MNCs were washed and exposed to 10 µg/ml dil-labeled acLDL (ThermoFisher) for 4 h at 37°C. LDL which was not taken up was removed by washing. Cells were exposed to CD32 antibody, stained for CD11b-FITC, washed and analyzed using a FACS Aria III (BD). Leukocyte counts measured in blood from the tail vein were used to calculate results for the respective cell populations as cell number/μl blood.

### Statistics

Statistical analyses were performed using GraphPad software. Data were tested for normal distribution using the Shapiro–Wilk normality test. Differences between groups were tested using Kruskal–Wallis test with Dunn’s multiple comparison test or one-way ANOVA with Tukey’s multiple comparison test. To determine the time ranges where the differences between groups were statistically significant, longitudinal *in vivo* microCT data for bone volume, bone formation, gap size and gap closing speed were modeled using Bayesian generalized linear mixed modeling in R (Team, 2015) package mgcv (Wood, 2006). The *p*-value for difference was extrapolated at every time point from the fitted curves and confidence intervals of the models. Correlations were calculated using Pearson’s correlation test. Data are presented as mean ± SEM.

## Results

### Kinetic of G-CSF Induced Cell Mobilization

Serum levels of human G-CSF reached a peak value at day 1 after the last injection with G-CSF (Figure 1B, *p* < 0.01). At day 5, the serum G-CSF concentration was not significant different from untreated control animals indicating a fast pharmacokinetic. Next, we examined the increase of the blood leukocyte count over time (Figure 1C). Significant elevated leukocyte counts were detected on the last day of G-CSF administration (day 0) and lasted for 10 days. At day 11, the leukocyte number decreased to the range of untreated animals. The peak in leukocyte accumulation was reached at day 1 with 7340 ± 299 leukocytes/μl blood which was significantly higher than the initial value of 4437 ± 125 leukocytes/μl (*p* < 0.001).

**Figure 2:**
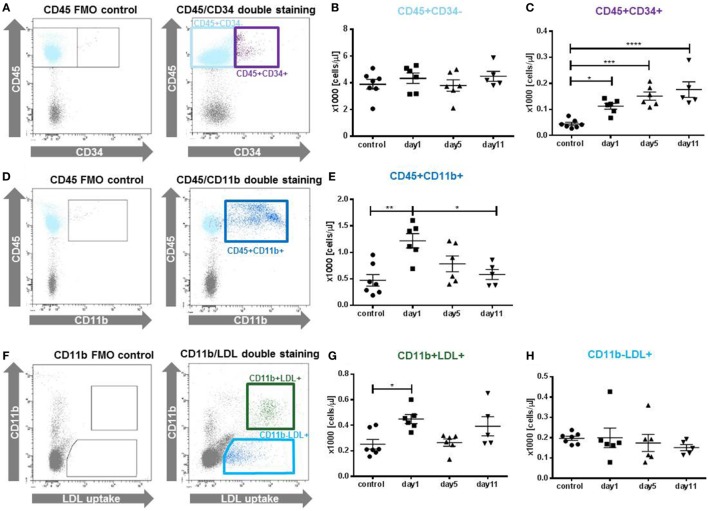
Cellular composition of the granulocyte colony-stimulating factor (G-CSF) mobilized cell population. Different cell populations were identified in peripheral blood mononuclear cells by antibody staining and flow cytometry at indicated time points after the last G-CSF injection. **(A)** Gating strategy for the identification of CD45+CD34+ and CD45+CD34− cells, gates were set in the CD45 single stained fluorescence minus one (FMO) control. **(B)** The fraction of CD45+CD34− was not affected. **(C)** Quantification of CD45+CD34+, representing hematopoietic progenitor cells. Cells were increased at all time points in comparison to untreated control animals. **(D)** Gating strategy for the identification of CD45+CD11b+ cells, including granulocytes, monocytes and macrophages. **(E)** CD45+CD11b+ cells showed a peak elevation at day 1. **(F)** Cells were incubated with Dil-acLDL particles to identify endocytotic active monocytes (CD11b+LDL+) and endothelial cells (CD11b−LDL+). **(G)** CD11b+LDL+ cells showed a peak increase at day1 compared to untreated control animals. **(H)** CD11b−LDL+ cells were not affected by the G-CSF treatment. Values are given as mean ± SEM. *p < 0.05; **p < 0.01; ***p < 0.001, ****p < 0.0001 calculated by one-way ANOVA with Tukey’s multiple comparison test **(B,C,E)**; and for non-normal distributed data **(G,H)** by Kruskal–Wallis test with Dunn’s multiple comparison test.

Analysis of different cell populations revealed a cell-specific kinetic of mobilization (Figure 2). While CD45+CD34− cells were not upregulated (Figure 2B), HPCs (CD45+CD34+) were significantly enriched at day 1 with 112 ± 27 cells/μl compared to 44 ± 6 cells/μl (*p* < 0.05) in control animals (Figure 2C). The accumulation of CD45+CD34+ cells further increased at day 5 and similarly high levels (*p* < 0.001) were detected at day 11. An upregulation was also detected for CD11b+leukocytes (Figures 2D,E). Here, the enrichment peaked at day 1, whereas at day 5 and day 11 no significant increase of CD11b+ cells was detected. Among the LDL+ cells, only the CD11b+ population was significant increased by G-CSF treatment (day 1: 449 ± 36 cells/μl vs. control: 252 ± 38 cells/μl, *p* < 0.05, Figure 2G), while no differences were observed for CD11b−LDL+ cells (Figure 2H).

### The Effect of G-CSF Induced Cell Mobilization on Bone Healing

**Figure 3:**
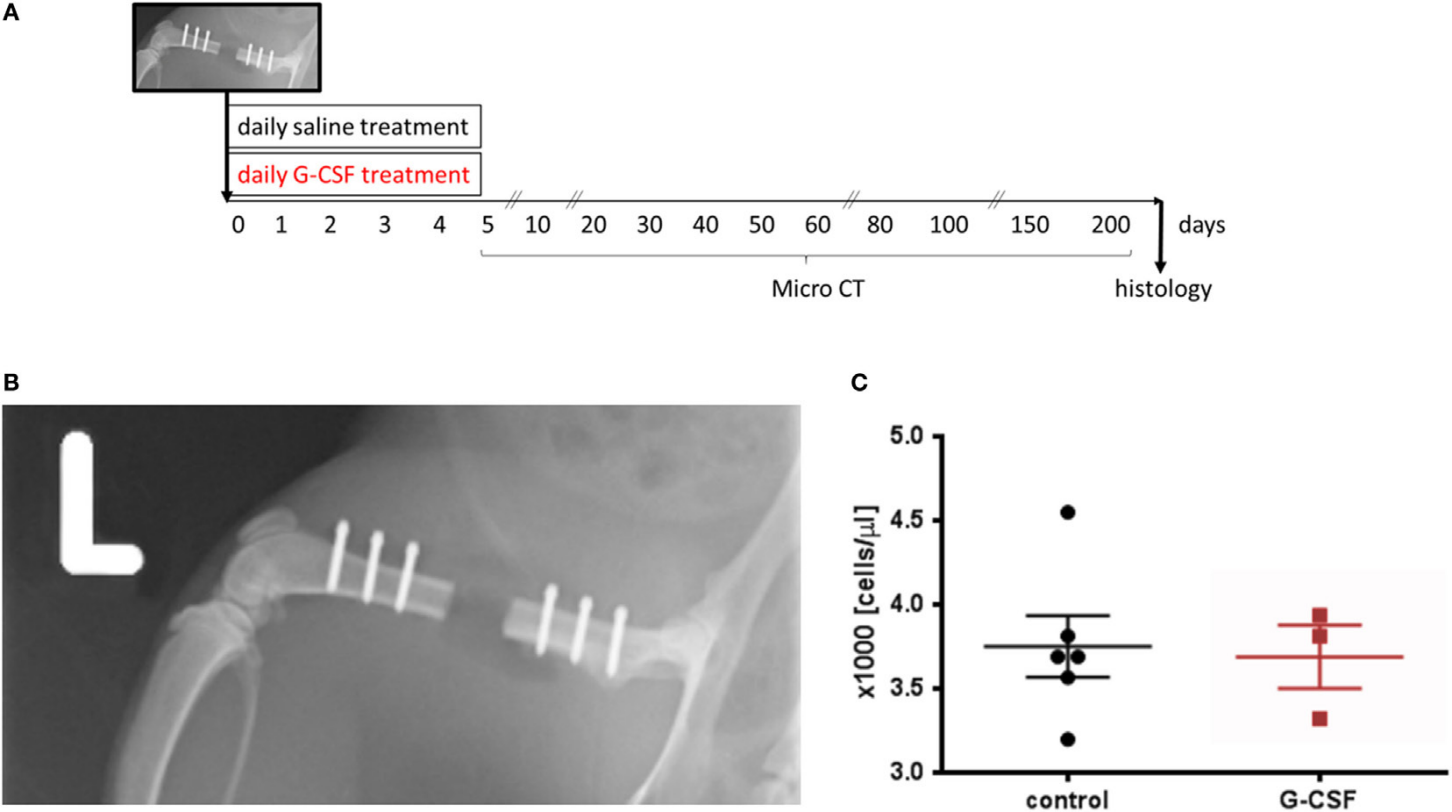
Granulocyte colony-stimulating factor treatment was tested in a femoral critical size bone defect model in rats. **(A)** Schematic presentation of the study design. **(B)** Postsurgery radiograph showing the 4.5 mm critical size osteotomy in the left femur. The defect was stabilized with a radiolucent PEEK plate using six screws. **(C)** Leukocyte counts upon surgery demonstrating similar leukocyte counts at the beginning of the study. Values are given as mean ± SEM.

We studied the influence of G-CSF treatment on the healing of a critical size bone defect in a femoral defect model in skeletally mature rats. Figure 3B shows a representative radiograph of the 4.5 mm defect fixed with a PEEK plate immediately after surgery. Blood sampling at surgery day showed that animals of both groups had similar leukocyte counts at the beginning of the study (Figure 3C).

**Figure 4:**
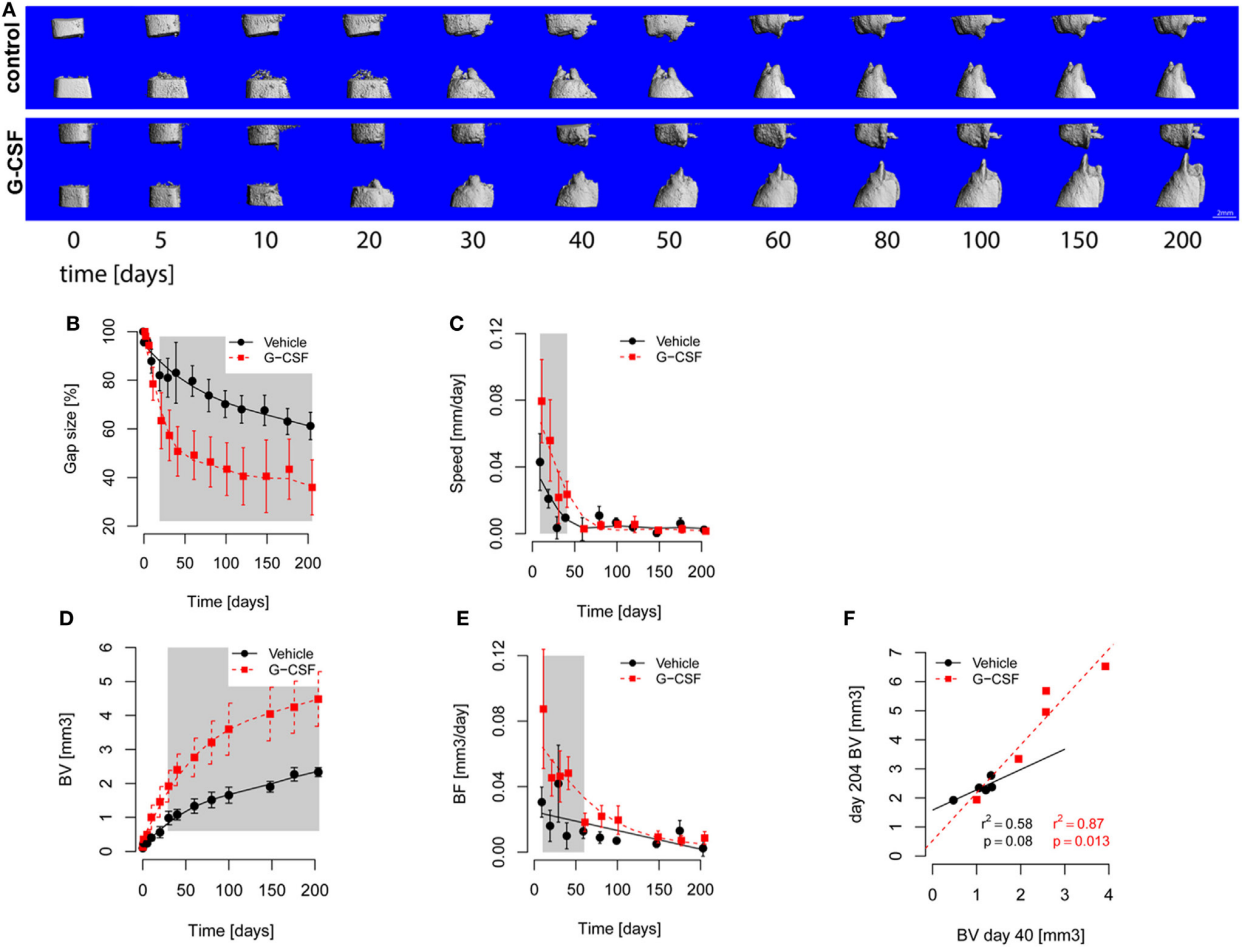
*In vivo* microcomputed tomography (microCT) monitoring of bone healing. The defects were scanned longitudinally over the course of the study. **(A)** 3D rendering of segmented microCT scans showing the progression of femoral defect healing. Bone formation into the defect was observed from both defect cuts already on day 5 postsurgery in all groups. All rats reached the end of the study with non-unions, but rats from the granulocyte colony-stimulating factor (G-CSF) group had smaller remaining gaps. The median rats of each group were picked for this figure. **(B–E)** Points are Mean ± SEM and curves show fitted models. The gray area shows *p* < 0.05 for significant differences between models. **(B)** Evolution of gap size over time. Gap size decreased in both groups with an inflection around day 30. Gap size was significantly smaller in G-CSF animals from day 20 on. **(C)** Reduction of gap size. The speed of gap size reduction was significantly higher in G-CSF (1.5 fold at beginning) from days 10 to 40. **(D)** Bone volume. Bone volume (BV) increased in both groups, showing an inflection around day 50, with G-CSF showing a faster increase in bone volume (resulting from higher bone formation). **(E)** Bone formation (BF, calculated from the difference in consecutive scans). Bone formation decreased in both groups over time. BF was significantly higher in G-CSF group from day 10 until day 60 (as high as twofold at day 10). **(F)** Correlation of bone volume at day 40 and day 204 (end of the study). The G-CSF-treated animals showed a higher correlation between day 40 and 204, suggesting that the effect of G-CSF treatment was already manifested at the early phase of healing.

Bone volume and density were longitudinally evaluated by microCT. Early bone formation into the defect was observed from both defect edges, forming conical-shaped in growth. Bone formation slowed down progressively over time and all rats finished the study with non-unions (Figure 4A). Analysis of the evolution of the gap size (Figure 4B) demonstrated a significant smaller gap size in G-CSF-treated animals starting from day 20. Next, we assessed the reduction in gap size per day and found a significant faster gap reduction in G-CSF-treated animals at early time points, namely between day 10 and 40 postsurgery (Figure 4C). In line with this, bone volume in control animals showed slower bone formation (day 211: 2.35 ± 0.14 mm^3^) compared to G-CSF-treated animals (4.45 ± 0.83 mm^3^, *p* < 0.01, Figure 4D). Again, analysis of the daily bone formation indicated a significant faster bone formation in G-CSF-treated animals from day 10 to day 60 (Figure 4E). These data suggested that G-CSF treatment would primarily affect the early stages of the healing process. Therefore, we examined the bone formation in the first 40 days after osteotomy in more detail. Bone formation at day 40 was significantly correlated with the result at the end of the study in G-CSF-treated animals (r^2^ = 0.87; *p* < 0.05) but not in control animals (Figure 4F).

In addition, the contralateral femoral midshafts were microCT scanned to investigate potential systemic effects of the G-CSF treatment. The cortical parameters (bone density, cortical thickness, moment of inertia) were identical between G-CSF and vehicle animals (data not shown). Furthermore, the bone formation outcomes from the defect site did not correlate with the contralateral bone parameters.

**Figure 5:**
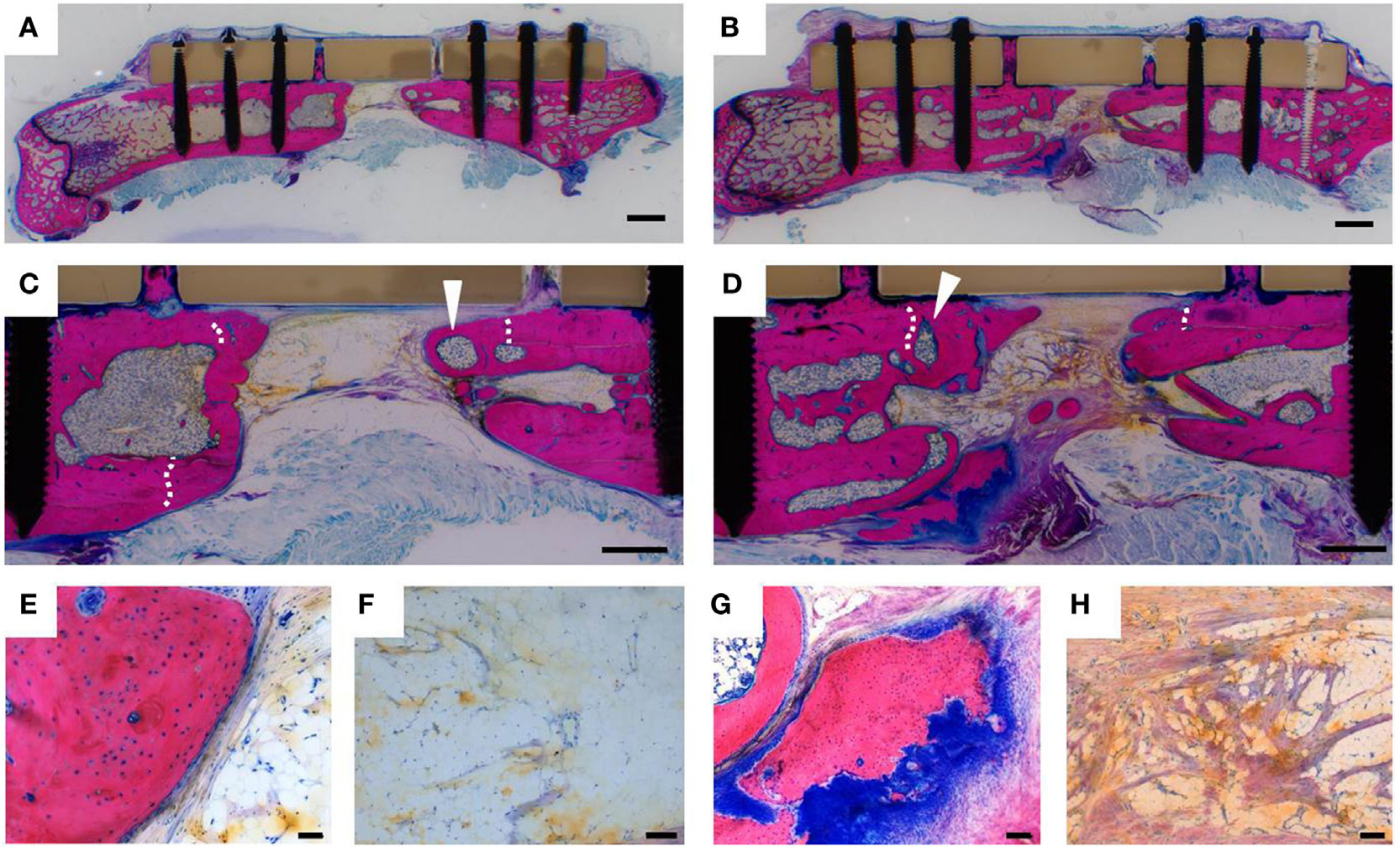
Histological analysis of bone healing. Representative Giemsa-Eosin stains of femurs dissected 230 days after surgery of a control animal **(A,C,E,F)** and a granulocyte colony-stimulating factor (G-CSF)-treated animal **(B,D,G,H)**. Bone formation occurred in animals of both experimental groups. White arrowheads show bone marrow cavities within the newly formed bone tissue. Osteotomy lines (white dashed lines) were still visible in the cortexes, indicating that more new bone has formed in the osteotomy gap in G-CSF-treated animals. High magnification images reveal that bone surfaces of control animals were lined by inactive flat osteoblasts **(E)** and the bone gap filled with vascularized fat tissue **(F)**. In G-CSF-treated animals, areas of active endochondral bone formation were detected by the dark blue glycosaminoglycan staining of cartilagenous tissue matrix **(G)**; collagen-rich fibrous tissue (purple) was detected in the bone gap **(H)**. Scale bars 2 mm **(A,B)**; 1 mm **(C,D)**; 100 µm **(F–H)**; 50 µm **(E)**.

The result of more efficient bone formation upon G-CSF treatment was confirmed by histology (Figure 5). In both experimental groups, new bone containing bone marrow cavities had formed, as indicated by the new tissue at the interior site of the osteotomy lines, which were still visible in cortexes (Figures 5C,D). In control animals, the remaining bone gap was predominantly filled with adipose tissue (Figures 5C,F), while in G-CSF-treated animals fibrous tissue rich in collagen fibers was observed (Figures 5D,H). Likewise, areas of active bone formation, indicated by strong blue staining of the cartilaginous tissue template (Figure 5G), were observed in the G-CSF group. In control animals, bone surfaces were mostly covered by flat, resting osteoblasts (Figure 5E).

## Discussion

### Mobilization of CD34+ Cells by G-CSF Administration

Despite its fast pharmacokinetic, G-CSF induced an elevation of blood leukocytes. The short serum half-life of injected G-CSF is in agreement with an earlier study reporting a half-life of 1.8–2.4 h for Neupogen (recombinant G-CSF) (Crobu et al., 2014). In this study, a peak accumulation of neutrophils appeared already on the second day of injections (Crobu et al., 2014). In another study, observing MNCs as well as CD34+ cells during 5 days of daily G-CSF injections, the highest values were detected on the last day of intramuscular injections (Deng et al., 2015). We detected the peak of total leukocyte accumulation one day after 5 consecutive days of sc G-CSF injections. Since we did not measure leukocyte counts during the G-CSF administration, it cannot be excluded that an even higher leukocyte mobilization was induced at earlier time points. However, CD45+CD34+ cells showed a continuous increase until day 11 suggesting that their mobilization peak indeed occurred at later time points. The variability in leukocyte mobilization kinetics between different studies is relatively minor considering their differences in doses, duration and G-CSF administration (Velders et al., 2002; Kong et al., 2004; Capoccia et al., 2006; Herbert et al., 2013; Deng et al., 2015; Teipel et al., 2015).

Our data suggest that the kinetics of cell mobilization varies in function of the leukocyte population (short peak mobilization of CD11b+ cells vs. long lasting mobilization of CD34+ cells), which might be explained by the different life span of the cell types. We also evaluated whether mature endothelial cells (CD11b−LDL+) would be targeted by G-CSF treatment. No mobilization of endothelial cells into the circulation was observed, but it is likely that G-CSF had a local effect on endothelial cells since various studies have reported a positive effect of G-CSF on proliferation and migration of endothelial cells (Bussolino et al., 1991; Park et al., 2008).

### The Role of Innate Immune Cells in Bone Healing

We have shown that CD11b+ innate immune cells were significantly increased by G-CSF administration reaching a peak level one day after the last injection. Stimulation of innate immune cells might affect the early proinflammatory phase of bone healing. A study of tibial fractures in mice demonstrated that infiltration of neutrophils and monocytes into the adjacent muscle was crucial for the healing process and blocking of cell recruitment led to impaired bone healing (Chan et al., 2015). It was also suggested that monocytes may be directly involved in bone formation by triggering the osteogenic differentiation of human MSCs (Omar et al., 2011). CD11b+ cells can also contribute to neovascularization. It was suggested that monocytes may represent a source of EPCs (Rehman et al., 2003) and it was proposed that G-CSF might change the gene expression profile of monocytes toward an increased expression of proangiogenic genes (Meier et al., 2013). Interestingly, in the context of hindlimb ischemia it was suggested that the proangiogenic effect after G-CSF treatment was rather mediated by monocytes than by incorporating EPCs (Capoccia et al., 2006) suggesting that also mobilized monocytes may have the ability to promote vascularization and by this bone healing. Future studies will be required to assess the underlying mechanisms which have led to the improved bone formation upon G-CSF treatment in the current study.

### Improved Bone Formation by G-CSF Injections

Our study clearly indicates that G-CSF promotes bone formation in a large segmental defect model. This is in line with earlier studies demonstrating beneficial effects of transplanted CD34+ cells, which were enriched by G-CSF mobilization from the bone marrow in some of the studies (Kuroda et al., 2014a; Kawakami et al., 2017). It was shown that systemically injected CD34+ cells have the potential to integrate into the newly formed tissue of a bone defect and to enhance vascularization two weeks after induction of a femoral fracture in nude rats, ultimately leading to improved bone healing (Matsumoto et al., 2006). In the current pilot study, we did not assess the early vascularization in the bone defect, but it can be assumed that the increased frequency of circulating CD34+ cells has here likewise promoted vascularization and bone formation. While this will be addressed in future studies, evaluation of bone formation over time by microCT revealed significantly faster bone formation in G-CSF-treated animals at early time points, starting at day 10 after induction of the bone defect. Based on our data on cell mobilization, this corresponds with the peak mobilization of CD34+ cells by G-CSF and therefore strongly suggests a clear relationship between cell mobilization and bone formation.

The aforementioned cell therapies are based on the transplantation of CD34+ cells, which is associated with several hurdles including low cell numbers, *ex vivo* manipulation of cells and immunogenicity. These disadvantages can be circumvented by direct application of G-CSF to mobilize endogenous cells in the same patient. The targeted approach in our current study has the additional benefit that G-CSF as well as some biosimilars are clinically approved, enabling fast translation of the treatment strategy into clinics. In the context of bone repair, it has been demonstrated that mobilization of EPCs into the circulation is part of the natural healing response (Mifune et al., 2008), indicating that such treatment follows the physiological healing process. Indeed, a preliminary clinical study has suggested beneficial effects of a G-CSF treatment on bone formation in patients undergoing high tibial valgus osteotomy with bone graft substitution (Marmotti et al., 2013). Cell mobilization by systemic G-CSF administration has also been applied in other fields with promising effects, including cardiovascular disease, wound healing and ischemic diseases (Fine et al., 2015; D’Amario et al., 2017; Huang et al., 2017). However, the clinical benefits are yet not fully clear and further investigations are required. Also, systemic administration of G-CSF may cause side effects, these might however be reduced by applying novel derivatives of G-CSF with longer half-life and higher mobilization efficiency (Misra et al., 2017).

Improved bone formation was also seen in several studies which have locally applied G-CSF to the defect site (Ishida et al., 2010; Minagawa et al., 2014; Liu et al., 2017a,b). In particular, Ishida et al. demonstrated an increased capillary density in the bone defect, detected after local G-CSF administration but also in a control group receiving sc G-CSF. With respect to these results it may be assumed that also in our study the G-CSF treatment promoted bone formation by enhancing early neo-vascularization. Nevertheless, future studies will be required to investigate the time course of vascularization through angiography during healing (Stewart et al., 2011) and to examine the role of mobilized cells herein, but this would require considerably more animals.

In this pilot study, we examined bone healing in a 4.5-mm femoral defect in skeletally mature rats. After internal fixation, this defect was left empty, and animals were solely treated with systemic application of G-CSF or saline. No bridging of the defects was observed in the current study. A defect size above 4 mm has been identified earlier as a critical size defect (Sato et al., 2014). Of note, the older age of the animals used in our study might have further slowed down the healing process and by this also prevented complete healing in the G-CSF treatment group (Histing et al., 2011). While the nature of the chosen animal model did not allow us to observe the effect of systemic G-CSF treatment on bone healing, it is yet a highly clinically relevant model.

### Perspectives

We have shown a positive influence of G-CSF induced cell mobilization on bone formation and gap reduction, although this treatment alone was not sufficient to heal a critical size bone defect in the current model. A possibility for improvement of the treatment protocol would be to elongate the serum accumulation of G-CSF, either by repeated injections or by using the PEGylated form of G-CSF which is significantly longer retained in the circulation (Arvedson et al., 2015). In addition, several strategies have been recently reported to improve the efficiency of cell mobilization, including the administration of Plerixafor, a clinically approved inhibitor of CXCR4 (Worel et al., 2016). Eventually, it will be interesting to combine G-CSF treatment with autologous, allogenic or synthetic bone graft material providing also mechanical support. Autologous bone grafts are still the gold standard treatment for critical size defects and non-unions, however associated with several drawbacks including donor site morbidity and limited availability. Allogeneic and synthetic grafts may overcome some of these drawbacks but are inferior in supporting vascularization. In this context, an additional G-CSF treatment may be advantageous. In conclusion, we here report that short time G-CSF administration significantly accelerated and promoted bone formation in a critical size bone defect. Since G-CSF is clinically approved, this is of major interest for the treatment of patients with critically-sized defects, non-unions and/or those individuals with a severe damage of defect-supplying vasculature.

## Ethics Statement

All animal experiments were approved by Graubünden Animal Commission (2014_30F; 2012_36) and performed in accordance with the Swiss Animal Protection Law in an Association for Assessment and Accreditation of Laboratory Animal Care Internal (AAALAC International) approved facility.

## Author Contributions

All authors have approved the final version of the manuscript and agree to be accountable for all aspects of the work in ensuring that questions related to the accuracy or integrity of any part of the work are appropriately investigated and resolved. The authors contributed to the work as follows: MHe and VS: design and conception of work, acquisition, analysis and interpretation of data, drafting the article, revision; SZ: design and conception of work, acquisition of data, revision; UE: design of work, acquisition of data, revision; MHi, KC, and UM: acquisition and interpretation of data, revision; MA: design and conception of work, interpretation of data, revision; SV: design and conception of work, acquisition and interpretation of data, revision; VS: design and conception of work, acquisition, analysis and interpretation of data, drafting the paper, revision.

## Conflict of Interest Statement

The authors declare that the research was conducted in the absence of any commercial or financial relationships that could be construed as a potential conflict of interest.
